# Geographic variation in human papillomavirus–related oropharyngeal cancer: Data from 4 multinational randomized trials

**DOI:** 10.1002/hed.24336

**Published:** 2016-01-08

**Authors:** Hisham Mehanna, Natalie Franklin, Natalie Compton, Max Robinson, Ned Powell, Nigel Biswas–Baldwin, Vindh Paleri, Andrew Hartley, Lydia Fresco, Hoda Al‐Booz, Elizabeth Junor, Iman El‐Hariry, Sally Roberts, Kevin Harrington, K. Kian Ang, Janet Dunn, Ciaran Woodman

**Affiliations:** ^1^ Institute of Head and Neck Studies and Education (InHANSE), School of Cancer SciencesUniversity of BirminghamEdgbastonBirminghamUnited Kingdom; ^2^ Roche Products Ltd, Shire ParkWelwyn Garden CityUnited Kingdom; ^3^ PPDGranta ParkGreat AbingtonCambridgeUnited Kingdom; ^4^ Centre for Oral Health ResearchNewcastle UniversityNewcastle‐upon‐TyneUnited Kingdom; ^5^ HPV Oncology Group, Institute of Cancer and Genetics, School of MedicineCardiff UniversityCardiffUnited Kingdom; ^6^ GSKUxbridgeMiddlesexUnited Kingdom; ^7^ Department of Otolaryngology–Head and Neck Surgery, Newcastle upon Tyne Hospitals, NHS Trust, and Northern Institute for Cancer ResearchNewcastle UniversityNewcastle upon TyneUnited Kingdom; ^8^ Hall‐Edwards Radiotherapy Research GroupOld Queen Elizabeth HospitalBirminghamUnited Kingdom; ^9^ Department of OncologyUniversity Hospitals Coventry and WarwickshireCoventryUnited Kingdom; ^10^ Bristol Haematology and Oncology CentreBristolUnited Kingdom; ^11^ Edinburgh Cancer CentreWestern General HospitalEdinburghUnited Kingdom; ^12^ Department of Clinical ResearchSynta Pharmaceuticals CorpLexingtonMassachusetts; ^13^ The Institute of Cancer ResearchLondonUnited Kingdom; ^14^ Department of Radiation OncologyThe University of Texas MD Anderson Cancer CenterHoustonTexas; ^15^ Warwick Clinical Trials UnitUniversity of WarwickGibbet Hill CampusCoventryUnited Kingdom; ^16^ School of Cancer SciencesUniversity of BirminghamEdgbastonBirminghamUnited Kingdom

**Keywords:** oropharynx, head and neck cancer, squamous cell carcinoma, human papillomavirus, prevalence

## Abstract

**Background:**

There are variations in the proportions of head and neck cancers caused by the human papillomavirus (HPV) between countries and regions. It is unclear if these are true variations or due to different study designs and assays.

**Methods:**

We tested formalin‐fixed paraffin‐embedded diagnostic biopsies for p16 immunohistochemistry and HPV‐DNA (by polymerase chain reaction [PCR] and in situ hybridization [ISH]) using validated protocols on samples from 801 patients with head and neck cancer recruited prospectively between 2006 and 2011 in 4 randomized controlled trials (RCTs).

**Results:**

Twenty‐one percent of patients (170 of 801) showed both HPV‐DNA and p16‐positivity, detected almost exclusively in oropharyngeal cancer (55%; 15 of 302); and only 1% of the patients (5 of 499) with nonoropharyngeal cancer were HPV positive. HPV‐positive oropharyngeal cancer differed between Western and Eastern Europe (37%, 155 of 422 vs 6%, 8 of 144; *p* < .0001) and between Western Europe and Asia (37% vs 2%; 4 of 217; *p* < .0001). Other independent determinants of HPV positivity were tumor site and smoking.

**Conclusion:**

This is the first study to establish geographic variability as an independent risk factor in HPV‐positive oropharyngeal cancer prevalence, with higher prevalence in Western Europe. © 2016 The Authors Head & Neck Published by Wiley Periodicals, Inc. *Head Neck* **38**: E1863–E1869, 2016

## INTRODUCTION

The proportion of head and neck cancers caused by the human papillomavirus (HPV) seems to have increased considerably over the past decade in several countries.^1^ However, there seems to be wide variations in the reported rates of both HPV‐positive oropharyngeal carcinoma and nonoropharyngeal cancer among countries and regions.^1^, ^2^ There are also little recent data available for Eastern Europe, Asia, or Africa.

It has become important to ascertain the regional differences in HPV‐positive head and neck cancer to understand the current trends and relative burden of HPV‐positive and HPV‐negative head and neck cancer. This will enable local clinicians and health commissioners to plan for the provision of health care, rehabilitative, and social support services for patients who are HPV‐positive who enjoy higher survival rates but have to deal with the considerable sequelae of their intensive treatment.^3^ It may also aid the assessment of the potential need for preventative public health measures; for example, the prophylactic vaccination of prepubescent boys, especially in regions with high HPV prevalence.^4^, ^5^ In addition, large phase III studies in head and neck cancer need to be global to facilitate timely recruitment and relevance across all licensing jurisdictions. For this, the relative distributions of HPV‐positive and HPV‐negative head and neck cancer in these areas must be known.

The variations in reported regional prevalence rates may be a reflection of regional differences in the profiles of established risk factors, such as primary site, smoking, or sexual habits,^6^ or as yet unknown factors. However, there remains concern that a significant part of the variability between geographic regions and between primary tumor sites may be due to study design biases, especially the use of different HPV detection techniques with little or no quality assurance,^2^, ^7^ the inclusion of retrospective cohorts recruited at different time periods,^1^, ^2^, ^8^ and misclassification of primary tumor sites.

In this study, our purposes were to overcome these potential deficiencies, to provide a recent and more accurate representation of the proportion of HPV‐positive head and neck cancer in different geographic regions, and to assess the role of region as a risk factor for HPV‐positive head and neck cancer. We compared the proportion of patients with HPV‐positive head and neck cancers and analyzed possible risk factors in a multinational cohort of patients with head and neck cancer who were all recruited prospectively in randomized controlled trials (RCTs), within a recent time frame. All samples were assessed for high‐risk HPV‐DNA using the same validated protocols in 2 quality‐assured laboratories.

## PATIENTS AND METHODS

### Patients

We examined formalin‐fixed paraffin‐embedded diagnostic biopsies from a total of 1049 patients with head and neck cancer who gave informed consent and were recruited to 4 multicenter RCTs: PET NECK trial (187 subjects), GSK EGF104334 (107 subjects), GSK EGF105884 (67 subjects), and GSK EGF102988 (688 subjects). Most subjects included in our study were recruited between 2007 and 2010.

### Ethical approval

Ethical approval for PET NECK trial (reference: 07/Q1604/35) was granted by the Oxfordshire Research Ethics Committee, May 2007. GSK EGF Ethical approval (reference: 06/MRE12/82) was granted by the Thames Valley Research Ethics Committee, March 2007.

The procedures followed were in accordance with the ethical standards of the committees on human experimentation and in accord with the Helsinki Declaration of 1975, as revised in 1983.

### Tissue samples, human papillomavirus, and p16 testing methods

Analysis was undertaken on tissue sections (4 μm) or on tissue microarrays (TMAs) with up to four 0.6‐mm diameter cores from each case. We took 10‐μm thick tissue curls from available blocks for DNA extraction and downstream polymerase chain reaction (PCR) analysis. All samples were analyzed for p16 expression by immunohistochemistry using a proprietary kit (CINtec Histology; MTM Laboratories AG, Germany) on a Benchmark Autostainer (Ventana Medical Systems, Sunnyvale, CA). Two pathologists (H.W. and M.R.) scored the test results independently, after undergoing calibration. Samples were assessed for high‐risk HPV‐DNA using a protocol based on that described and validated by Smeets et al.^9^ Samples were assessed by HPV in situ hybridization (ISH), using proprietary reagents (Inform HPV III Family 16 Probe (B); Ventana) on a Benchmark Autostainer (Ventana). For cases that were found to be p16‐positive/HPV‐negative by ISH using TMAs, whole tissue sections were tested to reduce misclassification as a consequence of possible sampling limitations inherent to TMA analysis. If the case remained p16‐positive/HPV‐negative by ISH on the whole tissue section, the case was then examined by consensus PCR‐enzyme immunoassay using a cocktail of probes for 14 high‐risk HPV types and separately with a cocktail of probes for 6 low‐risk types to control for the suboptimal sensitivity of HPV‐ISH compared to target amplification techniques.^9^ This latter protocol has been previously validated by our group and proven to have the same accuracy as using p16 and HPV‐PCR.^10^

For all samples in this study, cases that showed evidence of HPV‐DNA (either by ISH or consensus PCR) and showed high p16 expression (HPV‐positive/p16‐positive) were defined as “harboring biologically relevant oncogenic HPV infection.”^9^

### Statistical analysis

All subjects who were enrolled into the studies but who had not yet provided tumor tissue for biomarker testing were included in a summary of baseline and disease characteristics, but excluded from all other analyses. Subjects who had provided tumor tissue that was inadequate for testing were not included in the patient characteristics summaries. The latter are based on the subset of subjects who had valid results for both HPV and p16.

Subjects with at least one positive result for HPV and one positive result for p16 for any of the cores and sections were considered to be positive overall for that biomarker. If the patient had no positive cores or sections, then they were considered negative, otherwise their result was treated as unknown, and they were not included in the analysis. Subjects who tested negative for p16 and who had an inconclusive result for HPV were considered as HPV‐negative overall.

The analysis includes only subjects with valid results for both biomarkers (HPV‐DNA and p16). The demographic and baseline disease characteristics, including age, sex, primary tumor type, disease stage, smoking status, and geographic region, were summarized for subjects who had valid results for biomarkers, as well as those who did not provide tumor tissue for testing or who had insufficient tissue. The frequency and proportion of subjects testing HPV‐positive, p16‐positive, and those with combined positivity (HPV‐DNA‐positive and p16‐positive) were also reported by these characteristics. Where possible, odds ratios for combined positivity (HPV‐DNA‐positive and p16‐positive) were calculated for each characteristic, with suitable dichotomization where necessary. A logistic regression analysis was used to model the probability of testing positive for both HPV‐DNA and p16, adjusting for the risk factors listed above. Combined test results were grouped into 4 categories: (1) HPV‐positive/p16‐positive; (2) HPV‐positive/p16‐negative; (3) HPV‐negative/p16‐positive; and (4) p16‐negative/HPV‐negative, and summarized for patients with oropharyngeal cancer and nonoropharyngeal cancer tumors, as well as by geographic region.

## RESULTS

### Patient characteristics

A total of 1049 patients submitted samples, constituting 81% of total subjects recruited in all 4 studies at the time of the analysis. Eight hundred one patients had valid results for both biomarkers. The primary tumors included were squamous cell carcinomas originating from the oral cavity in 241 patients (30%), the oropharynx in 302 patients (38%), the larynx in 159 patients (20%), and the hypopharynx in 99 patients (12%). Compared to those who were included in the analysis, the cases that were not included had very similar baseline characteristics (see Table 1).

**Table 1:** Summary of demography and disease characteristics.

Variables	Subjects not tested for biomarkers (*n* = 248)	Subjects tested for biomarkers (*n* = 801)
Age, y		
Mean	55.8	54.3
SD	9.90	8.65
Median	56.0	55.0
Minimum	28	24
Maximum	80	81
Sex		
Female	45 (18%)	135 (17%)
Male	203 (82%)	666 (83%)
Primary tumor type		
Oral cavity	84 (34%)	241 (30%)
Oropharynx	80 (32%)	302 (38%)
Hypopharynx	36 (15%)	99 (12%)
Larynx	48 (19%)	159 (20%)
Stage		
II	5 (2%)	46 (6%)
III	47 (19%)	106 (13%)
IVa	179 (72%)	645 (81%)
IVb	17 (7%)	4 (<1%)
Geographic region		
Eastern Europe	15 (6%)	144 (18%)
Western Europe	143 (58%)	422 (53%)
Asia	71 (29%)	217 (27%)
South America	13 (5%)	14 (2%)
North America	6 (2%)	4 (<1%)

### Overall human papillomavirus‐DNA and p16 incidence

Of the 801 patients who had valid results for both p16 and HPV‐DNA, 193 (24%) showed HPV‐DNA (by ISH or PCR), 218 (27%) tested positive for p16, and 170 (21%) showed both HPV‐DNA and p16‐positivity (Supplementary Table S1, online only).

### Human papillomavirus prevalence by tumor site

There was a large difference in the distribution of HPV and p16‐positivity between tumor sites. Biologically relevant, oncogenic high‐risk HPV infection (HPV‐DNA‐positive/p16‐positive) was detected almost exclusively in oropharyngeal cancer (55%; 165 of 302 of oropharyngeal cases), whereas 1% (5 of 499) of nonoropharyngeal head and neck cancers were positive (Table 2 and Supplementary Table S1, online only).

**Table 2:** Human papillomavirus‐DNA and p16 incidence by age, sex, primary tumor type, smoking status, tumor stage, and geographic region.

	HPV‐DNA‐positive, no. of patients (%)	p16‐positive, no. of patients (%)	HPV‐DNA‐positive and p16‐positive, no. of patients (%)	OR for HPV‐positive (HPV‐DNA‐positive and p16‐positive)^a^
Age category				
≤50 y	45/236 (19)	52/236 (22)	42/236 (18)	0.74 (0.5, 1.09)
>50 y	148/565 (26)	166/565 (29)	128/565 (23)	Reference
Sex				
Male	156/666 (23)	173/666 (26)	137/666 (21)	Reference
Female	37/135 (27)	45/135 (33)	33/135 (24)	1.25 (0.81, 1.93)
Primary tumor site				
Oropharynx	173/302 (57)	186/302 (62)	165/302 (55)	Reference
Oral cavity	4/241 (2)	13/241 (5)	3/241 (1)	0.0105 (0.0033, 0.0334)
Larynx	10/159 (6)	13/159 (8)	2/159 (1)	0.0106 (0.0026, 0.0435)
Hypopharynx	6/99 (6)	6/99 (6)	0/99	
Smoking status				
Never smoked	55/166 (33)	59/166 (36)	50/166 (30)	Reference
Current smoker	37/178 (21)	39/178 (22)	28/178 (16)	0.43 (0.26, 0.73)
Former smoker	96/434 (22)	112/434 (26)	88/434 (20)	0.59 (0.39, 0.89)
Missing^b^	5/23 (22)	8/23 (35)	4/23 (17)	
Stage				
II	1/46 (2)	2/46 (4)	1/46 (2)	Reference
III	10/106 (9)	15/106 (14)	6/106 (6)	2.7 (0.3, 23.1)
IV(a + b)	182/649 (28)	201/649 (31)	163/649 (25)	15.1 (2.1, 110.4)
Region, all cases				
Eastern Europe	13/144 (9)	20/144 (14)	8/144 (6)	Reference
Western Europe	166/422 (39)	176/422 (42)	155/422 (37)	9.87 (4.71, 20.69)
Asia	10/217 (5)	18/217 (8)	4/217 (2)	0.32 (0.09, 1.08)
South America	3/14 (21)	3/14 (21)	2/14	Too few cases to analyze
North America	1/4 (25)	1/4 (25)	1/4	Too few cases to analyze
Region, oropharyngeal cancer only				
Eastern Europe	9/33 (27)	13/33 (39)	8/33 (24)	Reference
Western Europe	159/242 (66)	166/242 (69)	152/242 (63)	5.28 (2.28, 12.20)
Asia	2/23 (9)	4/23 (17)	2/23 (9)	0.30 (0.06, 1.56)
South America	2/2	2/2	2/2	Too few cases to analyze
North America	1/2	1/2	1/2	Too few cases to analyze

Abbreviations: HPV, human papillomavirus; OR, odds ratio.

Note: The denominators are the numbers of subjects tested for both biomarkers.

a HPV DNA positive means position on HPV DNA in‐situ hybridisation or HPV DNA PCR.

b Smoking status was not collected in study EGF104334.

### Prevalence by geographic location

The prevalence of HPV‐positivity in all cases of head and neck cancer was significantly lower in subjects from Asia (2%; 4 of 217) and Eastern Europe (6%; 8 of 144) compared to Western Europe (37%; 155 of 422; *p* < .0001 for both comparisons; Table 2).

Prevalence of biologically relevant oncogenic HPV (HPV‐DNA‐positive and p16‐positive) in oropharyngeal cancer also differed significantly by geographic location: 63% of the patients (152 of 242) in Western Europe, compared to 24% (8 of 33) in Eastern Europe (*p* < .0001) and 9% (2 of 23) in Asia (*p* = .0001; Table 2). For nonoropharyngeal head and neck cancer sites, HPV prevalence was very low (1%), and did not differ by region.

There were insufficient evaluable samples from North and South America to accurately comment on their incidence rates. There were also insufficient numbers to compare incidence rates of individual countries.

### Risk factors for human papillomavirus–positive head and neck cancer

**Figure 1:**
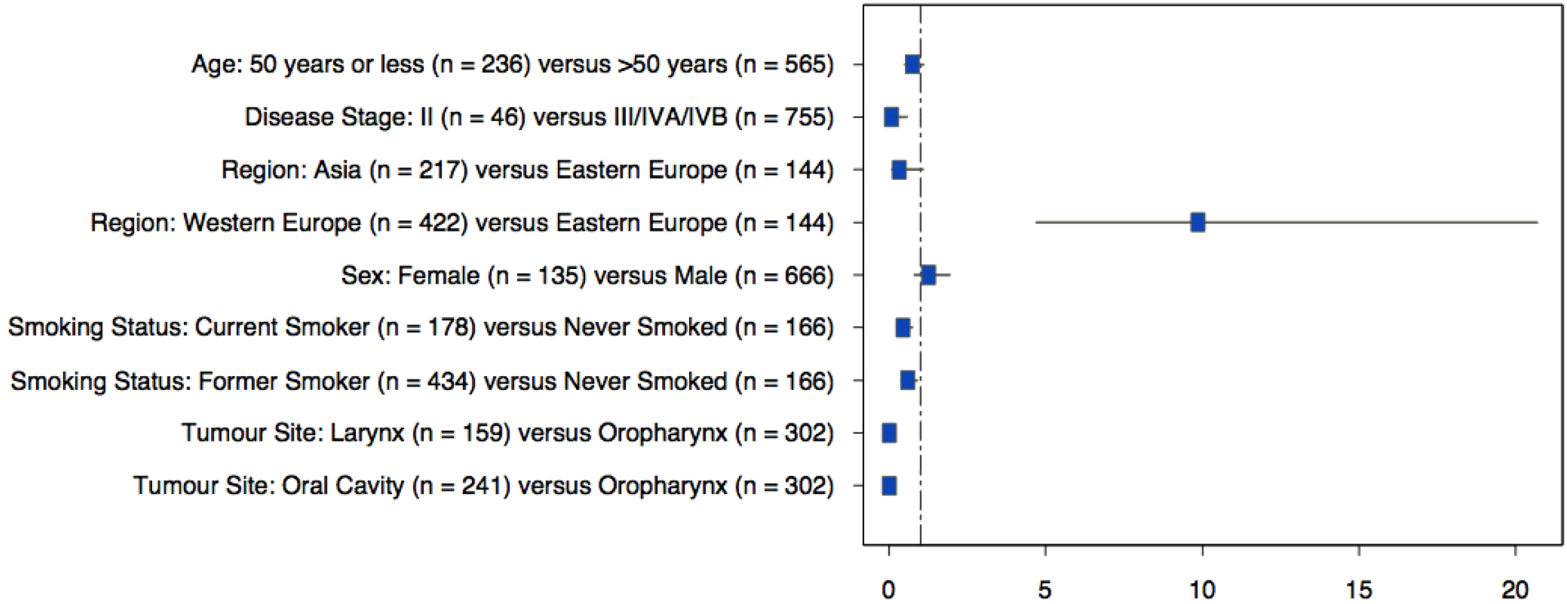
Forest plots of predictors of human papillomavirus (HPV) positivity for all subjects.

Factors significantly associated with HPV‐positive head and neck cancer, identified through univariate analysis, were primary site, stage of disease, smoking status, and geographic region. The overall odds of having HPV‐positive head and neck cancer in this 801 patient cohort was significantly lower in subjects with oral (odds ratio [OR] = 0.0105; 95% confidence interval [CI] = 0.003−0.033; *p* < .0001), and laryngeal cancers (OR = 0.0106; 95% CI = 0.003–0.044; *p* < .0001] compared to those with oropharyngeal cancer. The odds of having HPV‐positive cancer were also significantly lower for current smokers relative to nonsmokers (OR = 0.43; 95% CI = 0.26–0.73; *p* = .002) and for former smokers relative to nonsmokers (OR = 0.59; 95% CI = 0.39–0.89; *p* = .011). Subjects with stage IV disease were more likely to have HPV‐positive cancer (OR = 15.1; 95% CI = 2.1–110.4; *p* = .0115) compared to those with stage II disease. Importantly, subjects from Western Europe had a significantly higher risk of being HPV‐positive compared to those from Eastern Europe (OR = 9.9; 95% CI = 4.7–20.7; *p* < .0001; see Figure 1). There was a trend toward a lower risk of HPV‐positivity in subjects from Asia (OR = 0.32; 95% CI = 0.09–1.1; *p* = .067) compared to Eastern Europe, but this was not statistically significant. There was no association with age or sex (*p* = .13 and *p* = .32, respectively; Table 2).

**Figure 2:**
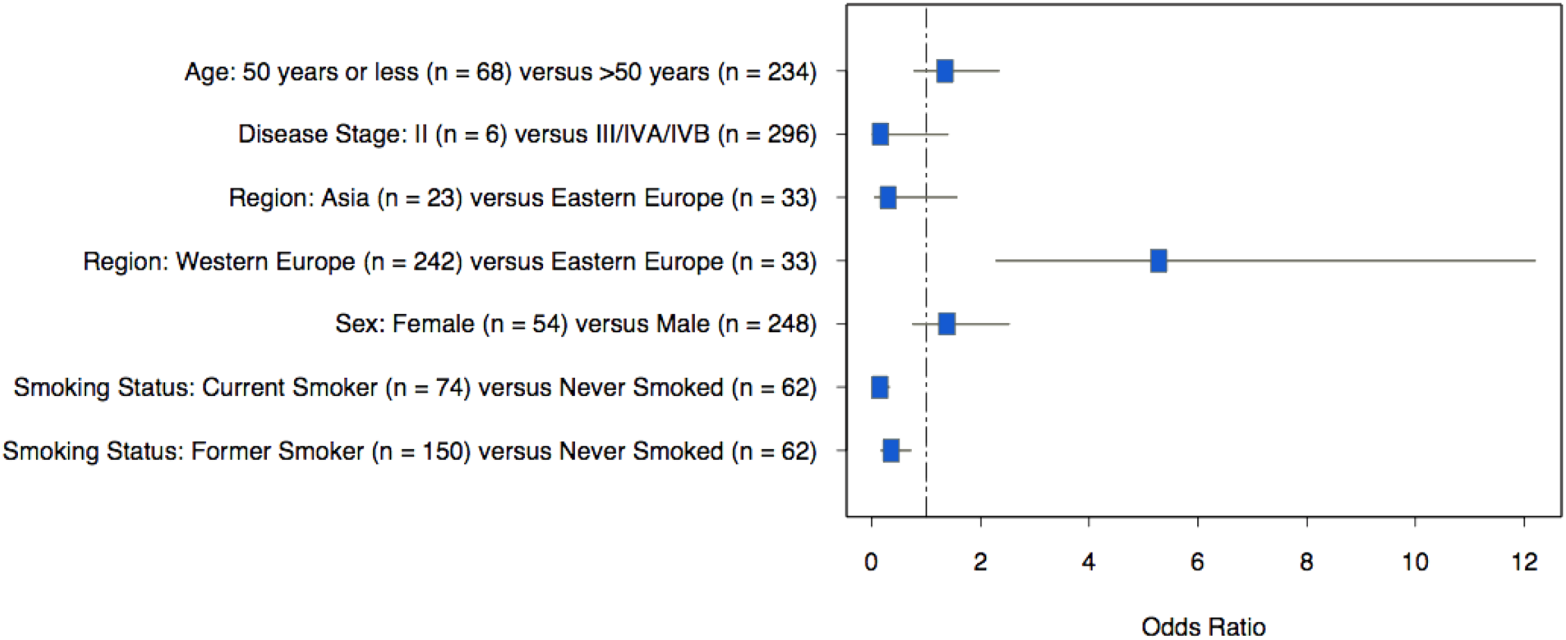
Forest plots of predictors of human papillomavirus (HPV) positivity for patients with oropharyngeal cancer only.

Considering only the 302 patients with oropharyngeal cancer, the odds of combined HPV‐DNA and p16‐positivity were again significantly lower for current smokers relative to nonsmokers (OR = 0.14; 95% CI = 0.07–0.31; *p* < .0001), for former smokers relative to nonsmokers (OR = 0.36; 95% CI = 0.18–0.71; *p* = .0035) and significantly higher for subjects from Western Europe (OR = 5.28; 95% CI = 2.28–12.20; *p* < .0001) compared to those from Eastern Europe (see Figure 2). Again, there was a trend toward a lower likelihood of HPV‐positivity in oropharyngeal cancer subjects from Asia (OR = 0.3; 95% CI = 0.06–1.56; p = .1511) compared to Eastern Europe, but this did not reach statistical significance. The odds for HPV‐positive oropharyngeal cancer were not significantly associated with age (OR for those aged ≤50 years vs those aged >50 years = 1.35; 95% CI = 0.78–2.33; *p* = .29), or sex (OR for women relative to men = 1.38; 95% CI = 0.8–2.5; *p* = .29), or disease stage (OR for stage II vs other = 0.16; 95% CI = 0.02–1.39; *p* = .097 see Figure 2).

Multivariate analysis using a logistic regression model was performed, first with inclusion of all potential risk factors (age, sex, primary site, clinical stage, smoking status, and geographic region), and second using stepwise selection. Only primary site, smoking status, and geographic region were found to have a significant influence on the probability of biologically relevant oncogenic HPV infection (HPV‐DNA and p16‐positivity) in head and neck cancer (Table 3 and Supplementary Table S2, online only).

**Table 3:** Multivariate analyses of association of human papillomavirus positivity for all subjects using a model without selection.

Risk factor	Coefficient, β (SE)	*p* value	OR for combined HPV/p16 positivity (95% CI)
Intercept	−0.827 (1.583)	.60	–
Smoking status			
Never smoked, reference	–	–	1
Current	−2.02 (0.43)	< .0001	0.133 (0.06–0.31)
Former	−1.39 (0.38)	.0003	0.250 (0.12–0.53)
Sex			
Male, reference group	–	–	1
Female	−0.07 (0.34)	.83	0.93 (0.475–1.818)
Region			
Eastern Europe, reference	–	–	1
Asia	−0.71 (0.77)	.36	0.49 (0.11–2.23)
Western Europe	1.91 (0.46)	< .0001	6.74 (2.72–16.72)
Tumor site			
Oropharyngeal, reference	–	–	1
Nonoropharyngeal	−4.38 (0.48)	< .0001	0.013 (0.005–0.03)
Disease stage			
II, reference group	–	–	1
III/IV	1.55 (1.33)	.25	4.70 (0.35–63.9)
Age, y	−0.01 (0.02)	.45	0.99 (0.96–1.02)

Abbreviations: OR, odds ratio; HPV, human papillomavirus; 95% CI, 95% confidence interval.

The model using a stepwise logistic regression model is included as Supplementary Table S2, online only.

Age, sex, primary site, stage, smoking status, and region were introduced into the model.

All subjects, considering sex, disease stage, age, region (Eastern/Western Europe and Asia), tumor site, and smoking status without selection. Similar findings were observed when analyses were repeated for oropharyngeal cancer subjects only (Supplementary Table S3, online only).

When examining risk factors for oropharyngeal cancer alone, multivariate analysis revealed only smoking status and geographic region to be significantly associated with combined HPV‐positivity and p16‐positivity (Supplementary Table S3, online only). This was the same whether the factors were introduced together or in a stepwise fashion. There was no statistically significant interaction between the risk factors (*p* = .69).

## DISCUSSION

This study demonstrated large, significant differences in the proportion of HPV‐positive oropharyngeal cancer among Western Europe, Eastern Europe, and Asia, with HPV‐associated head and neck cancer being predominantly a disease of the oropharynx in Western Europe. Geographic location seems to have a strong association with HPV‐positivity in oropharyngeal cancer, regardless of smoking status, which is a well‐established risk factor. To the best of our knowledge, this is the first time that a study firmly has established geographic variability in the prevalence of HPV‐positive oropharyngeal cancer. The study highlights the need for ascertainment of local rates before deciding on local health policies and strategies related to oropharyngeal cancer. The results also demonstrate the very low, relative prevalences of HPV in nonoropharyngeal cancer tumors, and emphasize that, despite the current interest in HPV‐related disease, the prevalence of HPV‐negative head and neck cancer in many countries remains considerably higher than that of HPV‐positive disease.

By collating one of the largest cohorts ever collected, using prospective multinational trials, and analyzing them using the same assay and methodology, this study has been able to overcome some of the main limitations of previous studies. After accounting for tumor site and smoking status, geographic region has a significant influence on the probability of testing positive for HPV. The reasons for these geographic variations remain speculative. A likely factor may be related to differences in the prevalence rates of oral HPV infection.^10^ and the underlying various sexual practices, particularly oral sex and multiple sexual partners, which have been shown to be risk factors for oral HPV infection and HPV‐positive oropharyngeal cancer^6^, ^11^, ^12^, ^13^ It would be of interest to collect sexual history in future trials. Other factors that could explain these regional factors may be related to biological and genetic differences in the population studied. This holds true for other cancers; for example, in cervical cancer (also caused by HPV), genetic differences may determine susceptibility to HPV infection,^14^ and/or transformation and progression of premalignant lesions to cancer.^15^ Importantly, however, because of our study design, methodological and assay variations can be eliminated as the potential main explanation for the wide geographic differences in HPV‐positivity rates.

Some hypothesize that smoking may protect against the development of HPV‐positive oropharyngeal cancer.^16^ As smoking status is significantly associated with HPV‐positive oropharyngeal cancer rates (Tables 2 and 3), one important potential explanation for regional variability in HPV‐positive oropharyngeal cancer rates may be differences in smoking behavior. Western European smoking rates are thought to have decreased in recent years. An association with smoking behavior could suggest that, as smoking rates decrease in Eastern Europe and Asia, HPV‐positive oropharyngeal cancer rates may increase to the levels of Western Europe. However, our study shows that region continues to be an independent risk factor, even after adjustment for smoking in the multivariate analysis. Furthermore, analysis of reported national smoking rates shows that although Eastern Europe has the highest reported national cigarette consumption, China has similar rates and India has much lower reported national cigarette consumption compared to Western Europe.^17^

This study had some limitations. Only 76% of eligible subjects had evaluable samples. Although this is high when compared to similar studies, it may have still introduced bias to our results. The subjects included had been recruited within trials with eligibility criteria, including having treatment for curative intent. This introduces the possibility of bias compared to population‐based estimates, which may also include subjects with very early disease or those undergoing palliative treatment, who are more likely to have HPV‐negative oropharyngeal cancer.^18^ However, because the eligibility criteria were applied consistently across all the recruiting countries, the relative proportions of HPV‐positivity and, hence, ORs between regions are unlikely to change significantly. Finally, there were lower numbers of cases included from Eastern Europe and Asia compared to Western Europe. There were also insufficient samples from the different countries to allow definitive comparisons between countries of the same geographic region.

Our study also enabled the quantification of the relative prevalence of HPV in nonoropharyngeal head and neck cancer compared to oropharyngeal cancer, reports of which have varied widely in the literature.^1^ The prevalence rate of HPV in nonoropharyngeal head and neck cancer in our study was much lower than that reported in previous, usually small retrospective studies.^2^ Our results seem to be consistent with the low levels of HPV prevalence in nonoropharyngeal head and neck cancer reported by the larger studies (eg, 1.6% by Scapoli et al).^19^

Our findings have implications for the planning of future clinical trials. It is clear that HPV‐positive and HPV‐negative head and neck cancers have different characteristics and survival rates, and are thus different disease entities.^20^ As such, the need for collaboration of national collaborative groups will become even more important because of the fragmentation and stratification of patient populations. By enabling more precise estimates of regional prevalence of HPV‐positivity, the data in this study will help improve the accuracy of designing and planning global trials in HPV‐positive and HPV‐negative head and neck cancer.

The findings of this study also have significant implications for local health care policies. In regions where HPV‐positive disease is prevalent, it will be necessary to consider and plan for the long‐term support and survivorship needs of these younger subjects, who are much more likely to survive their cancer, but who will also have to deal with the long‐term effects on them and their families of their disease and treatment.

Over three fourths of the subjects recruited in our study had HPV‐negative head and neck cancer (Table 2). Although there is a lot of interest currently in HPV‐positive head and neck cancer, there is still a considerably higher burden of HPV‐negative head and neck cancer globally. These subjects have a much worse prognosis than their HPV‐positive counterparts. More effective treatment regimens are much needed to improve outcomes of treatment for this large patient group.

Finally, the significant differences in the prevalence of HPV‐positive head and neck cancer have implications for preventative public health measures, especially in resource‐limited settings. The cost‐effectiveness of the HPV vaccination for prepubescent boys is highly dependent on the prevalence of the disease, among other factors. In areas of low HPV prevalence, especially in countries with low health care expenditure, resource and efforts may be best directed toward smoking cessation policies.

## Supporting information

Supporting Information
