# Supplementary material for: Geographic variation in human papillomavirus–related oropharyngeal cancer: Data from 4 multinational randomized trials
Source: Head Neck. 2016 Jan 8;38(Suppl 1):E1863–9. doi: 10.1002/hed.24336 (PMC4869674; doi:10.1002/hed.24336)
Supplement: Supplementary file 1 — Supporting Information [file HED-38-E1863-s001.docx]

Supplementary tables

Supplementary Table S1 - Summary of HPV DNA (on ISH or PCR) and p16 Status by Tumour Site, Excluding Subjects with Unknown Result for Either HPV or p16 or unknown primary site

| **Tumour Site** | **Oropharyngeal**  ***N=302*Number (%)** | **Non-oropharyngeal**  ***N=499***  **Number (%)** | **Total**  ***N=801***  **Number (%)** |
| --- | --- | --- | --- |
| **HPV DNA Positive, p16 Positive** | 165 (55%) | 5 (1%) | 170 (21%) |
| **HPV DNA Positive, p16 Negative** | 8 (3%) | 15 (3%) | 23 (3%) |
| **HPV DNA Negative, p16 Positive** | 21 (7%) | 27 (5%) | 48 (6%) |
| **HPV DNA Negative, p16 Negative** | 108 (36%) | 452 (91%) | 560 (70%) |

Note: Subjects with no HPV result but a negative p16 result are included as HPV Negative, P16 negative.

Supplementary Table S2:

All subjects, considering gender, disease stage, age, region (Eastern/Western Europe and Asia), tumour site and smoking status *with stepwise selection.* Those factors that were found to be insignificant were removed. Those that remained significant are shown below.

| **Risk Factor** | **Coefficient (β)**  **(Standard Error)** | ***P-*value** | **Odds ratio for combined HPV/p16 positivity**  **(95% CI)** |
| --- | --- | --- | --- |
| **Intercept** | -0.15 (0.48) | 0.76 | - |
| **Smoking Status:**  **Never Smoked (Reference)**  **Current**  **Former** | -  -1.96 (0.42)  -1.33 (0.37) | -  <0.0001  0.0004 | 1  0.14 (0.06,0.32)  0·26 (0.13,0.55) |
| **Region:**  **Eastern Europe (Reference)**  **Asia**  **Western Europe** | -  -0.57 (0.77)  2.01 (0.45) | -  0.45  <.0001 | 1  0.56 (0.13,2.53)  7.47 (3.07,18.17) |
| **Tumour Site:**  **Oropharyngeal (Reference)**  **Non-oropharyngeal** | -  -4.36 (0.48) | -  <0.0001 | 1  0·013(0.005,0.03) |

Supplementary Table S3:

Multivariate analyses of predictors of HPV positivity for OPC subjects only using both a model without selection and using a stepwise logistic regression model

The risk factors - age, gender, stage, smoking status and region - were introduced into the model in a stepwise fashion. Those that were found to be insignificant in the presence of other factors were removed. Those that were significant remained and are shown below.

1. OPC Subjects only, considering gender, disease stage, age, region (Eastern/Western Europe and Asia) and smoking status *without selection*

| **Risk Factor** | **Coefficient (β)**  **(Standard Error)** | **p-value** | **Odds ratio for combined HPV/p16 positivity**  **(95% CI)** |
| --- | --- | --- | --- |
| Intercept | -0.700 (1.654) | 0.6721 | - |
| Smoking Status:    Never Smoked (Reference)    Current    Former | -  -2.248 (0.475)  -1.504 (0.434) | -  <0.0001  0.0005 | 1  0.106 (0.042,0.268)  0.222 (0.095,0.520) |
| Gender:  Male (Reference)  Female | -  0.040 (0.367) | -  0.9134 | 1  1.041 (0.507,2.135) |
| Region:  Eastern Europe (Reference)  Asia  Western Europe | -  -1.915 (1.158)  1.861 (0.487) | -  0.0981  0.0001 | 1  0.147 (0.015,1.424)  6.428 (2.474,16.70) |
| Disease Stage  II (Reference)  III/IV | -  1.593 (1.389) | -  0.2516 | 1  4.918 (0.323,74.87) |
| Age (yrs) | -0.012 (0.018) | 0.4903 | 0.988 (0.954,1.023) |

(b). OPC Subjects only, considering gender, disease stage, age, region (Eastern/Western Europe and Asia) and smoking status *with stepwise selection*

| **Risk Factor** | **Coefficient (β)**  **(Standard Error)** | **p-value** | **Odds ratio for combined HPV/p16 positivity**  **(95% CI)** |
| --- | --- | --- | --- |
| Intercept | 0.048 (0.505) | 0.9237 | - |
| Smoking Status:    Never Smoked (Reference)    Current    Former | -  -2.198 (0.462)  -1.455 (0.421) | -  <0.0001  0.0006 | 1  0.111 (0.045,0.275)  0.233 (0.102,0.533) |
| Region:  Eastern Europe (Reference)  Asia  Western Europe | -  -1.750 (1.148)  1.977 (0.474) | -  0.1275  <.0001 | 1  0.174 (0.018,1.649)  7.218 (2.853,18.26) |

Note: Tumour site is grouped into oropharyngeal versus non-oropharyngeal as there were too few subjects in each subgroup to perform valid analyses separately.
